# The Integration of Transcriptome and Metabolome Analyses Provides Insights into the Determinants of the Wood Properties in *Toona ciliata*

**DOI:** 10.3390/ijms25084541

**Published:** 2024-04-21

**Authors:** Zhi Wang, Jinsong Wu, Weijia Kong, Yu Zhou, Chunyi Ye, Qianyun Yuan, Yongjia Zhang, Pei Li

**Affiliations:** 1 College of Forestry and Landscape Architecture, South China Agricultural University, Guangzhou 510642, China; 13956496597@163.com (Z.W.); 19065335411@163.com (J.W.); 18085096832@163.com (W.K.); 15687213051@163.com (Y.Z.); 19120530656@163.com (C.Y.); erissss@126.com (Q.Y.); z1213558516@163.com (Y.Z.); 2 Guangdong Key Laboratory for Innovative Development and Utilization of Forest Plant Germplasm, Guangzhou 510642, China

**Keywords:** *Toona ciliata*, wood properties, transcriptomic, metabolomic, lignin

## Abstract

*Toona ciliata*, also known as Chinese mahogany, is a high-quality and fast-growing wood species with a high economic value. The wood properties of *T. ciliata* of different provenances vary significantly. In this study, we conducted comprehensive transcriptome and metabolome analyses of red and non-red *T. ciliata* wood cores of different provenances to compare their wood properties and explore the differential metabolites and genes that govern the variation in their wood properties. Through combined analyses, three differential genes and two metabolites were identified that are possibly related to lignin synthesis. The lignin content in wood cores from *T. ciliata* of different provenances shows significant variation following systematic measurement and comparisons. The gene Tci09G002190, one of the three differential genes, was identified as a member of the *CAD* (Cinnamyl alcohol dehydrogenase) gene family of *T. ciliata*, which is associated with lignin synthesis. Our data provide insights into the determinants of the wood properties in *T. ciliata*, providing a solid foundation for research into the subsequent mechanisms of the formation of *T. ciliata* wood.

## 1. Introduction

*Toona ciliata*, belonging to the *Toona* order of the Meliaceae family, is an endangered plant with key protection status in China and is listed in the reference list of principal cultivated precious tree species [1]. As one of the precious fast-growing trees, *T. ciliata* is now an important species for plantation and use in Southern China [2]. Its wood has an attractive texture with russet hues and a straight shape when it is dried; it is also soft and corrosion-resistant with good machinability, making it of high economic value [3]. *T. ciliata* is marketed at home and abroad and is known as Chinese mahogany [2]. Previous research on *T. ciliata* breeding found that the wood of the species is not always dark reddish-brown in color, and that its wood basic density and fiber morphology show significant differences; the color of the wood from certain provenances of *T. ciliata* can be lighter or the red color may not be not obvious, with wood that is less dense and fibers that are fine and short. Other provenances, however, have high-quality wood that is darker in color, with higher basic density and fibers that are thick and long [4]. Clarification of the differences among provenances in the wood properties of *T. ciliata* and investigation of the mechanisms of wood formation would provide a solid basis for the selection and breeding of this species.

The main aim of metabolomics is to quantify the multiple dynamic responses of living organisms to external stimuli, pathophysiological changes, and their own genetic mutations at the metabolite level [5,6]. The introduction and development of this technique has provided a more efficient method of identifying metabolites in plants and elucidating the metabolic pathways regarding their synthesis in plants [7,8]. Transcriptome sequencing can obtain almost all the transcripts and gene sequences of an assigned cell or tissues of a species in an assigned state; the results can then be used to study the level of gene expression, the gene function, the gene structure, variable splicing, and the prediction of new transcripts [9]. Wood, as a natural polymeric compound, is mainly composed of cellulose, hemicellulose, lignin, and extractives [10]. And the wood’s color, basic density, odor, strength, and other factors will affect the wood’s properties [11,12,13]. At the same time, the color of wood and other wood properties are affected by many secondary metabolites. Consequently, the transcriptome and metabolome play an important role in the study of wood properties. In this study, under a comprehensive analysis of the metabolome and transcriptome, it was found that there are differential metabolites in the lignin synthesis pathway, and they are significantly related to differentially expressed genes, which provides a new direction for research.

Lignin, as a cell wall component and one of the three main components of plant secondary walls, provides mechanical support and water conduction within the vascular system. Studies have demonstrated that lignin can absorb light at up to 500 nm and is an important source of color production in wood [14], with different tree species having different organizational structures and functional groups [15]. The synthesis of lignin involves three pathways: the shikimic acid pathway, the phenylpropionic acid pathway, and the specific pathway, and involves many enzymes and monomers. And in these pathways, there will also be many small molecular substances or products that will directly or indirectly affect the color of the wood [16,17,18]. Understanding the synthesis mechanism of lignin is crucial for the study of wood properties.

In this study, different metabolites and genes were screened in red and non-red *T. ciliata* wood cores. Significant differences in the wood properties were observed following a combined analysis of the transcriptome and metabolome. We also analyzed the relationship between the metabolites and genes, exploring the reasons for the differences in the wood properties of *T. ciliata*. Our research lays a solid foundation for future research into the mechanism of *T. ciliata* wood formation.

## 2. Results

### 2.1. Metabolome Analysis

**Figure 1:**
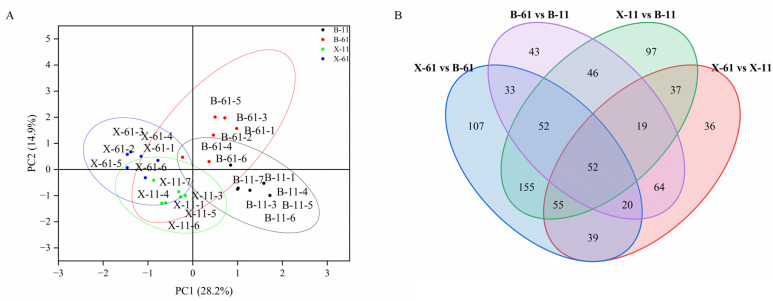
(**A**) Three-dimensional plot of PCA for difference grouping; (**B**) Wayne’s plot of differential metabolites across groups, Different colours represent different groupings, Overlapping colors represent metabolites shared by two or more groups.

**Figure 2:**
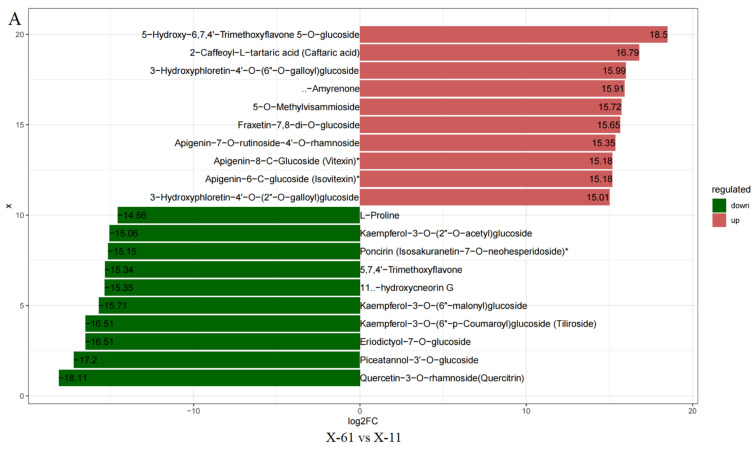

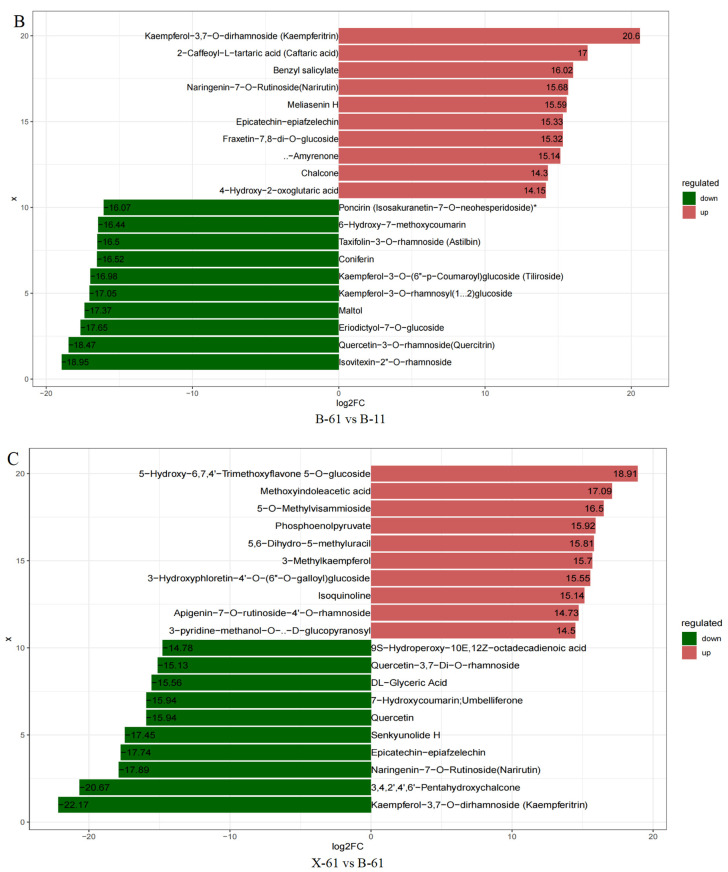

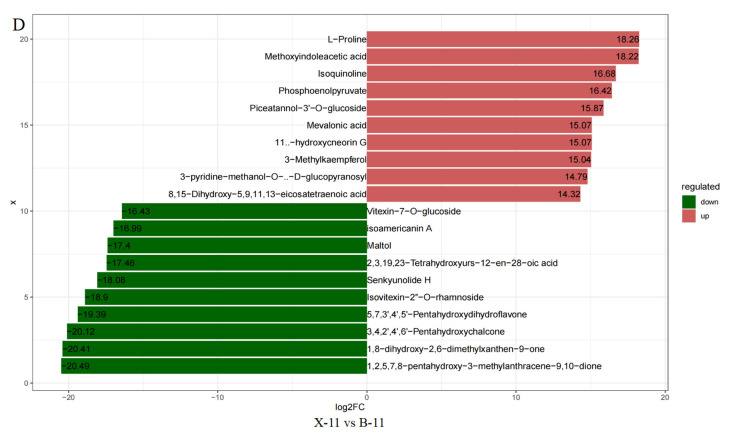
Differential multiplicity histogram. Note: (**A**–**D)** are the different groups. Right: the label of each bar indicates the name of the metabolite differentiated by upregulation (red) and downregulation (green); the length of the bar represents logFC.

**Figure 3:**
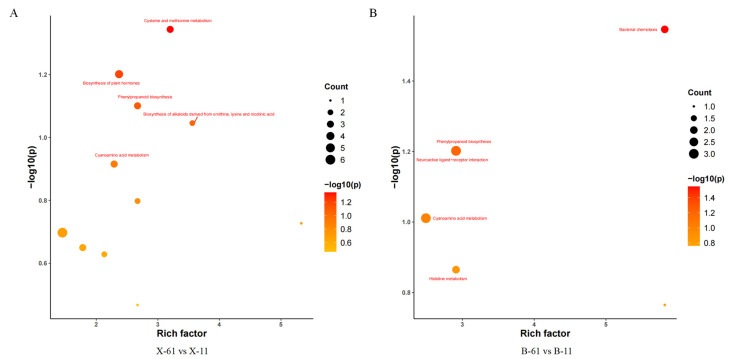

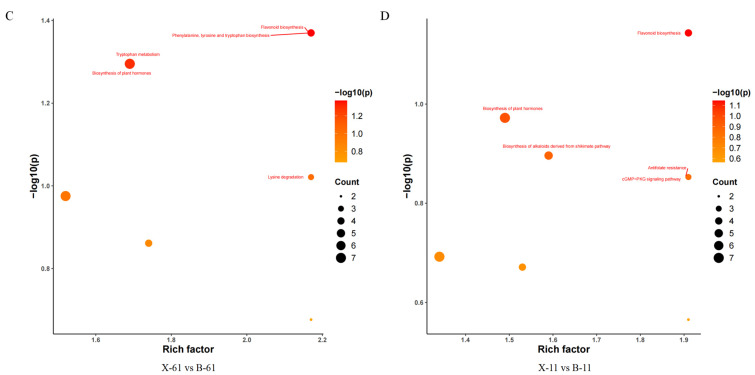
Differential metabolite KEGG enrichment factor bubble plot. Note: (**A**–**D**) are the different groups. The *x*-axis is the enrichment factor of the differential metabolites enriched in that pathway, the *y*-axis is the pathway *p*-value, and the size of the dots represents the number of enriched differential metabolites.

The principal component analysis (PCA) results indicated a high similarity between samples within groups and a low similarity between samples across groups (Figure 1A). A total of 52 differential metabolites were common across all groups (Figure 1B). In total, 322, 329, 513, and 522 differential metabolites were identified in groups X-61 vs. X-11, B-61 vs. B-11, X-61 vs. B-61, and X-11 vs. B-11, respectively. Among these, upregulated and downregulated differential metabolites are 157, 135, 307, and 299 and 165, 194, 206, and 223 in different groups, respectively (Figure 2 and Figure S1). Metabolic analyses were performed on each pair of the four groups, revealing significant differences in the metabolites involved in the phenylpropanoid biosynthesis pathway (Figure 3). The final metabolites of this pathway were observed to be the three types of lignin monomers (H-lignin, G-lignin, and S-lignin). These results suggest that the wood properties of *T. ciliata* vary and that differences in color may be related to the lignin content of the wood.

### 2.2. Transcriptome Analysis

#### 2.2.1. Transcriptome Sequencing Results

**Figure 4:**
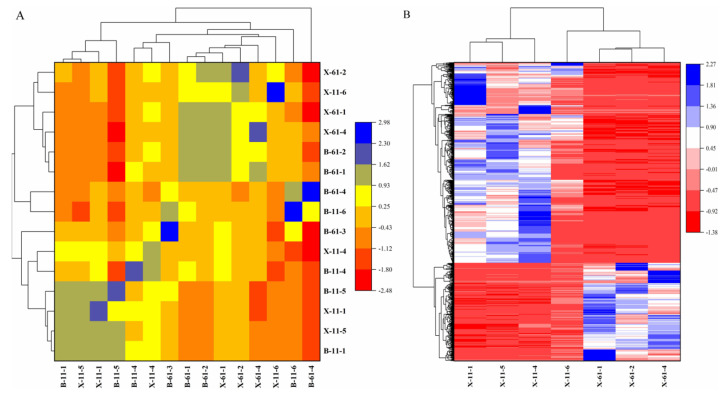
(**A**) Heatmap of expression correlation between two−by−two samples; (**B**) clustering of differentially expressed genes.

A transcriptome analysis was conducted on all samples, resulting in a total of 97.12 GB of clean data. Each sample contained 5.75 GB of clean data and a Q30 base percentage of 92.55% or higher. The clean reads of each sample were compared with the designated reference genome for sequence comparison. The comparison efficiency ranged from 80.54% to 94.24%. From the comparison results, variable splicing prediction and gene structure optimization analysis were performed. This resulted in the discovery of 1692 new genes, of which 593 were functionally annotated. Using a fold change ≥ 2 and an FDR < 0.01 as criteria for differential gene screening, results were obtained for differentially expressed gene lists, differentially expressed gene functional enrichment analyses, GSEA analyses, differential variable splicing, and differential gene–protein interactions in each comparison group (Figure 4).

#### 2.2.2. Differential Gene Results

**Figure 5:**
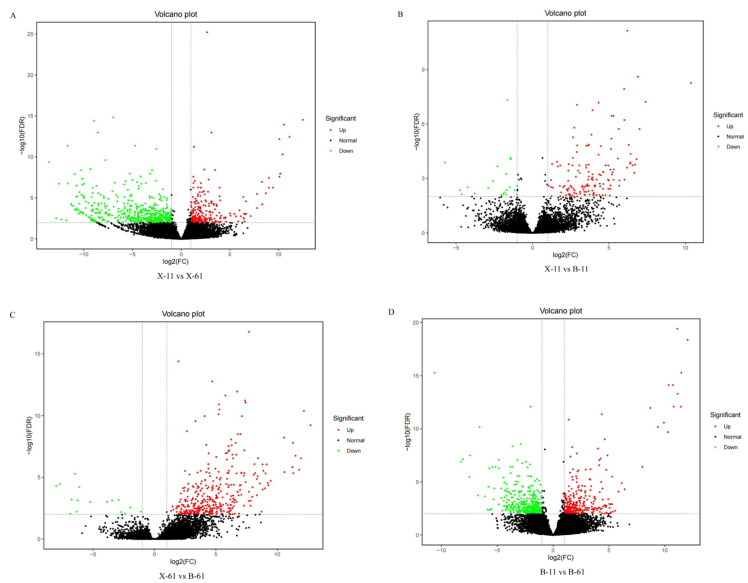
Differential expression volcano plots: (**A**) X-11 vs. B-11; (**B**) X-61 vs. B-61; (**C**) X-11 vs. X-61; (**D**) B-11 vs. B-61.

Differentially expressed genes were identified using differential analysis software with screening based on the gene count value in each sample. The screening criteria used to identify differentially expressed genes were a fold change ≥ 2 and an FDR < 0.01. Using the X-11 sample as the control group, 734 differential genes were identified, with 241 upregulated and 493 downregulated genes in the X-61 sample. And 162 differential genes were identified, with 141 upregulated genes and 21 downregulated genes in the B-11 sample compared with the X-11 sample (X-11 vs. B-11). We compared the gene expression of the B-61 sample with that of the control group, the X-61 sample: 384 differential genes were identified, and 368 were upregulated while 16 were downregulated. Finally, a total of 705 differentially expressed genes were identified, with 299 genes upregulated and 406 genes downregulated in the B-61 sample when compared with the B-11 sample (B-11 vs. B-61) (Figure 5).

#### 2.2.3. Differential Gene Pathway Analysis

**Figure 6:**
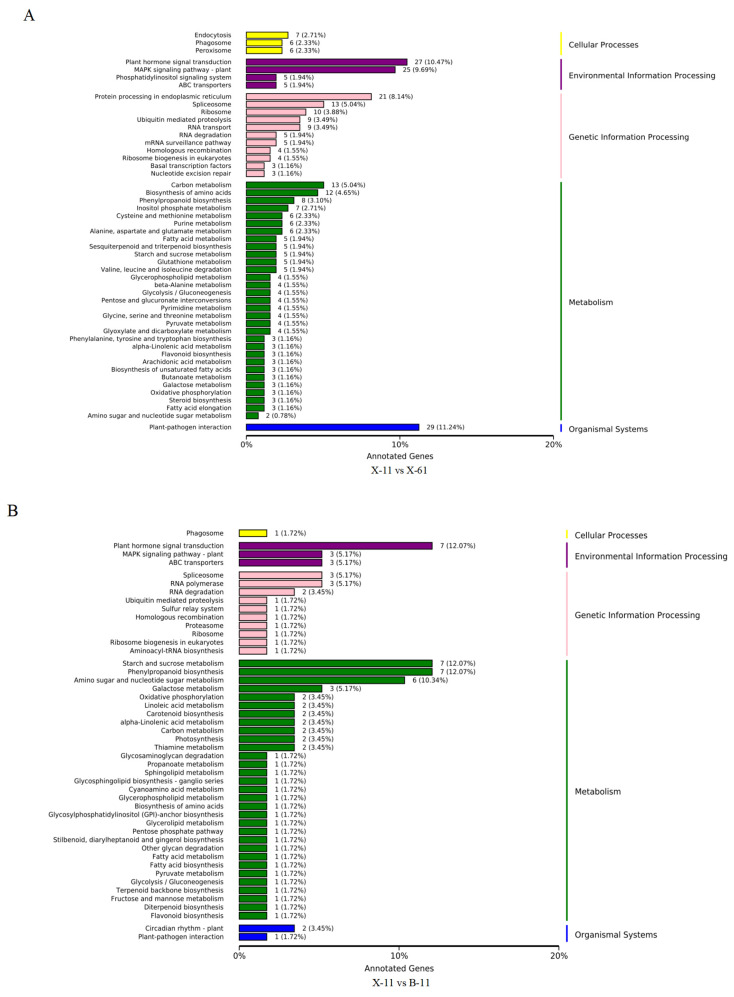

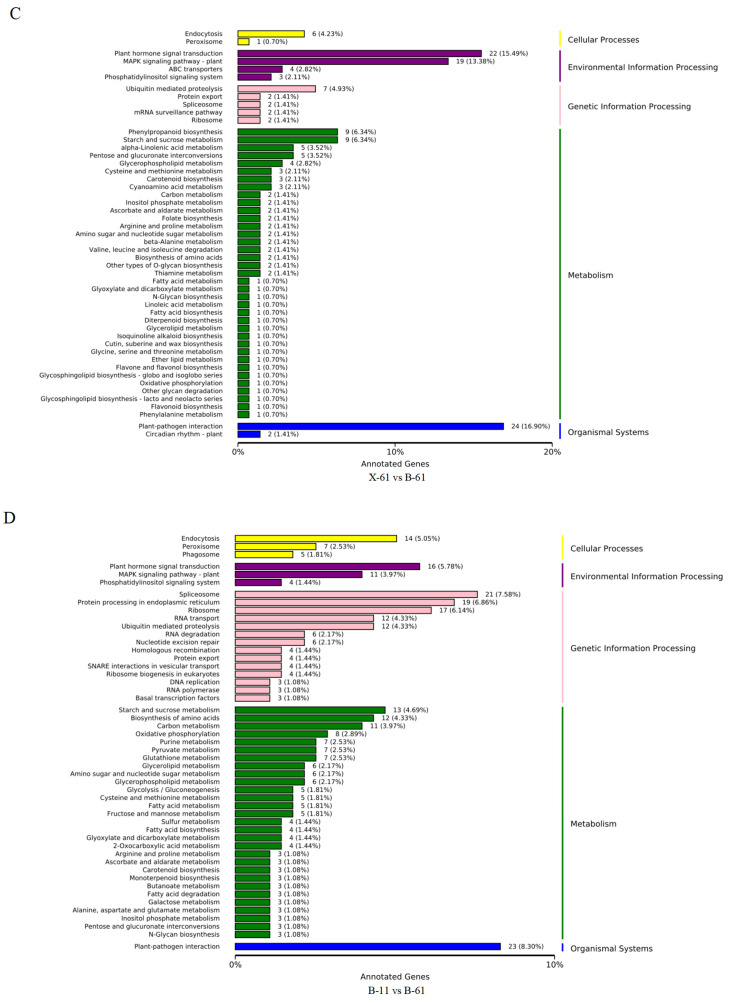
KEGG classification map of differentially expressed genes.(**A**) X-11 vs. X-61; (**B**) X-11 vs. B-11; (**C**) X-61 vs. B-61; (**D**) B-11 vs. B-61.

**Figure 7:**
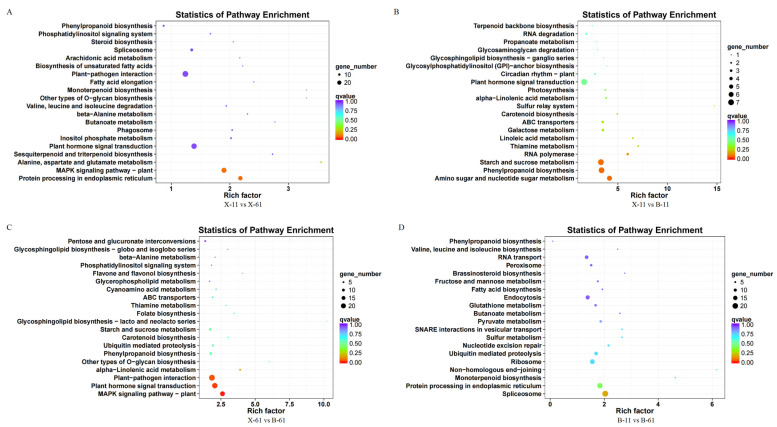
KEGG pathway enrichment scatter plot of differentially expressed genes.(**A**) X-11 vs. X-61; (**B**) X-11 vs. B-11; (**C**) X-61 vs. B-61; (**D**) B-11 vs. B-61.

We enriched the pathways of the genes by comparing the heartwood or sapwood of the red and non-red groups (X-61, B-61, X-11, and B-11) to determine the role of differentially expressed genes in lignin synthesis. We then categorized the annotation results of the differentially expressed genes according to the types of pathways in the KEGG database. These differentially expressed genes were classified into five major categories: cellular processes, environmental information processing, genetic information processing, metabolism, and organismal systems. Most of those involved in the information processing, genetic information processing, metabolism, and organismal system categories were annotated under the pathways of the three major categories of environmental information processing, genetic information processing, and metabolism (Figure 6). There were eight differentially expressed genes in the pathway of phenylpropanoid biosynthesis (ko00940) in group X-61 vs. X-11 (Figure 7A). In group X-11 vs. B-11, the three most significantly varied pathways, with the lowest Q value, were amino sugar and nucleotide sugar metabolism (ko00520), phenylpropanoid biosynthesis (ko00940), and starch and sucrose metabolism (ko00500). The enriched pathway related to lignin synthesis was phenylpropanoid biosynthesis (ko00940), which is associated with seven differentially expressed genes (Figure 7B). There were eight differentially expressed genes in the phenylpropanoid biosynthesis (ko00940) pathway, which is related to lignin synthesis in group X-61 vs. B-61 (Figure 7C). Thus, we concluded that the genes that were differently expressed in the phenylpropanoid biosynthesis (ko00940) pathway might play an important role in lignin synthesis.

### 2.3. Comprehensive Analysis of the Metabolome and Transcriptome

**Figure 8:**
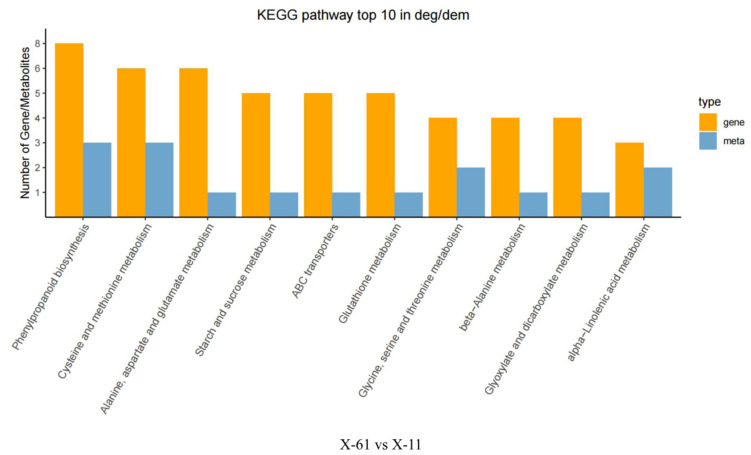
Top 10 pathways containing the most differential genes (yellow bar)/differential metabolites (blue bar).

**Figure 9:**
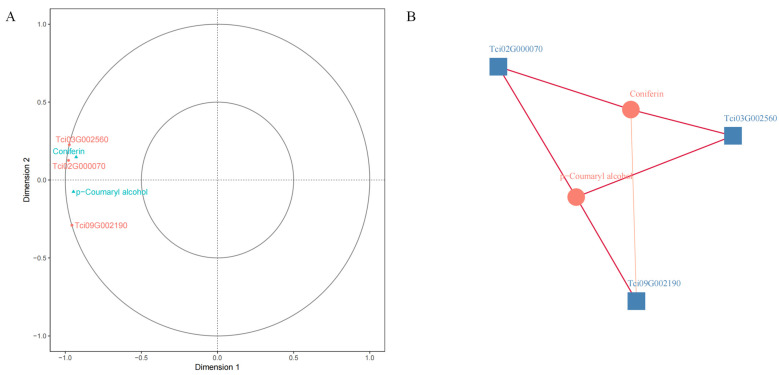
(**A**) Typical correlation analysis plot. Note: The four regions are distinguished by crosses in the figure and within the same region; the further the distance from the origin, the higher the correlation, and the higher the correlation between metabolites and genes that are closer in the same quadrant. The angle formed by the line connecting the gene and metabolite to the origin reflects the magnitude of the correlation; an acute angle represents a positive correlation, and an obtuse angle represents a negative correlation. (**B**) Correlation network diagram. Circles are differential metabolites, and squares are differential genes. The red line shows a positive correlation.

We identified 23 metabolic pathways in which differential genes and differential metabolites were jointly involved in X-61 and X-11 using comprehensive analysis of the transcriptome and metabolome. Among all these pathways, phenylpropanoid biosynthesis (ko00940) has the most differentially expressed genes and metabolites, with eight differential genes and three differential metabolites (Figure 8). The correlations between the differential genes and metabolites were analyzed, revealing that three differential genes (Tci03G002560, Tci02G000070, and Tci09G002190) were correlated with two metabolites (p-coumaryl alcohol and coniferin). The rest of the differential genes and metabolites have a correlation coefficient greater than 0.9 (representing a significant level), except for the correlation coefficients between Tci09G002190 and coniferin, among which Tci09G002190 and p-coumaryl have the most significant correlation (Figure 9).

Thus, we chose Tci09G002190 as the target for the subsequent studies. The gene sequence of the transcriptome was compared with the reference sequence from NCBI. We observed that Tci09G002190 significantly overlaps with the *CAD* (cinnamyl alcohol dehydrogenase) gene sequence. Then, we analyzed the conserved structural domains and found that the protein encoded by the *CAD* gene contained conserved structural domains of ADH_N and ADH_zinc_N. CAD is a key enzyme in lignin synthesis; hence, we hypothesized that Tci09G002190 is related to lignin synthesis in *T. ciliata.*

### 2.4. qRT-PCR Verification

**Figure 10:**
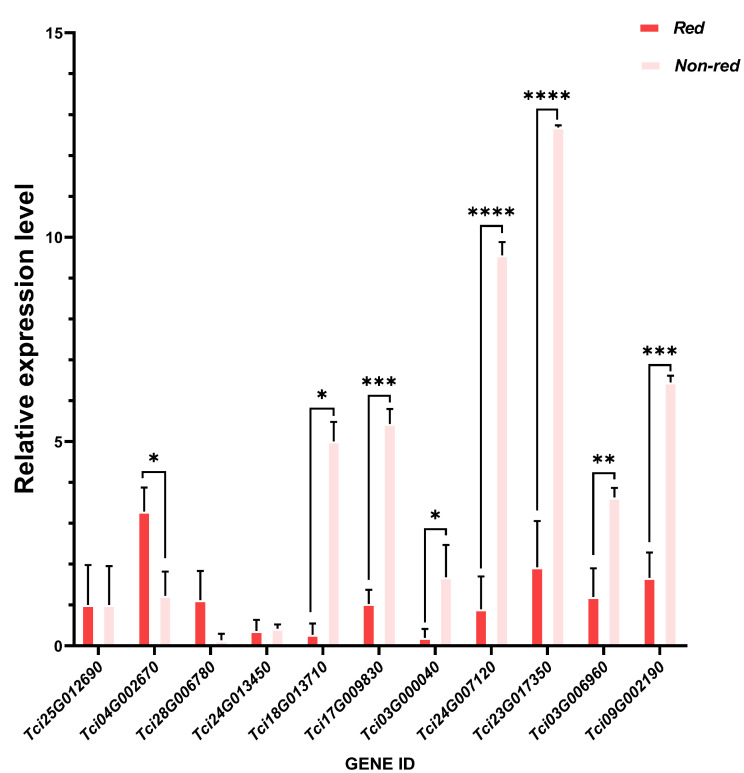
qRT-PCR validation results. Note: Values are means ± SD of three independent biological replicates. Asterisks denote Student’s *t*-test significance: * *p* < 0.05, ** *p* < 0.01, *** *p* < 0.001, and **** *p* < 0.0001.

The non-red and red wood cores were set as the control and experimental group, and five genes that were significantly upregulated and downregulated were screened based on the results of differential ploidy of genes, counts, FDR (false discovery rate), etc., respectively (Table 1). Figure 10 demonstrates that the downregulated and upregulated genes are associated with high expression in the non-red and red groups, respectively. The downregulated genes also had a higher expression in the red group, confirming the transcriptome results.

### 2.5. Measurement of Lignin Content

**Figure 11:**
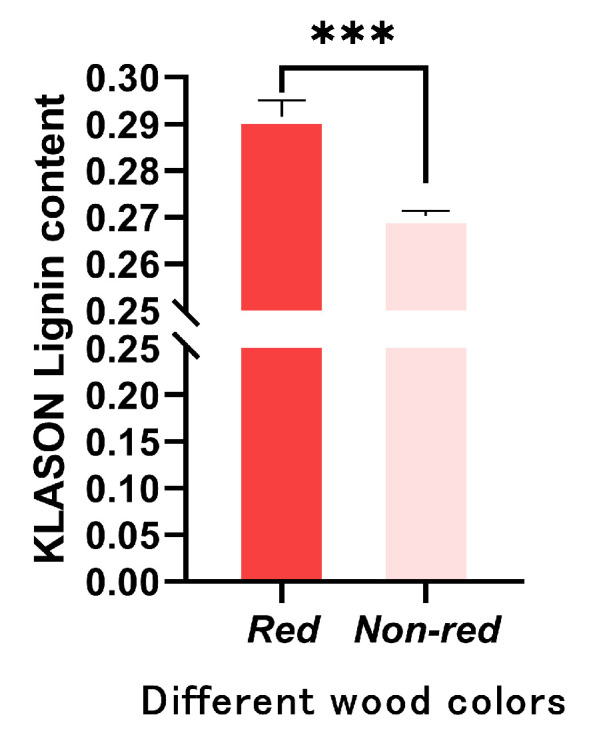
KALASON lignin content in different wood colors. Note: Values are means ±SD of three independent biological replicates. Asterisks denote Student’s *t*-test significance: *** *p* < 0.001.

The red wood cores were divided into two groups—red wood cores and non-red wood cores—measured according to the lignin determination method. The results are presented in Figure 8. The lignin content of the red wood cores (29.16%) was higher than that of the non-red wood cores (27.03%). This indicated that the color of *T. ciliata* wood could be affected by the lignin content to an extent (Figure 11).

### 2.6. Screening of Key Genes in Lignin Synthesis

**Figure 12:**
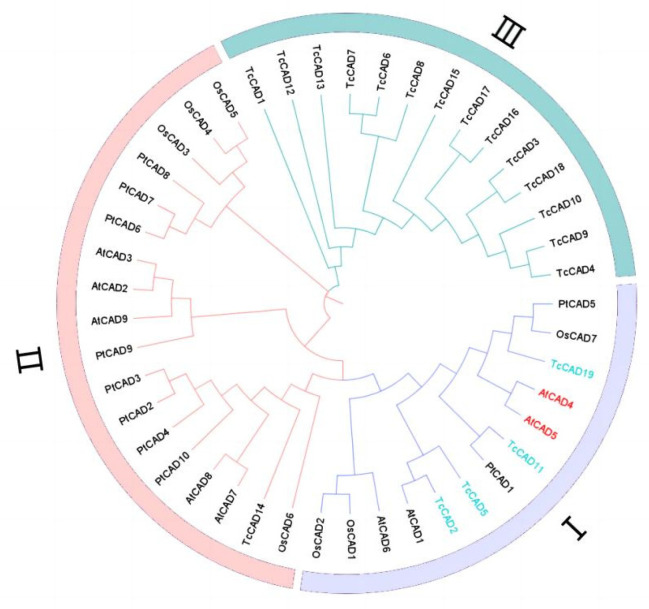
Phylogenetic tree of *CAD* gene families in *T. ciliata*, *A. thaliana*, *Populus trichocarpa*, and *O. sativa*.

From our comprehensive analysis of the metabolome and transcriptome, combined with the measurement of lignin content, we posited that lignin synthesis may be a key factor affecting the differences in the wood properties of *T. ciliata*. The differential gene Tci09G002190 was chosen for analysis. The sequence was screened from the transcriptome sequencing results, and a homology comparison of amino acid sequences was performed using NCBI. And 19 *TcCADs* were identified within the whole genome sequence of *T. ciliata* using TBtools. The 19 *TcCAD* genes were renamed, changing from *TcCAD1* to *TcCAD19*, according to their distribution on different chromosomes. To further analyze the phylogenetic relationship of *TcCAD* genes in *T. ciliata*, the protein sequences of the *CAD* gene families of *A. thaliana* (9), *O. sativa* (7), and *Populus trichocarpa* (10) obtained from previous studies were subjected to multiple sequence alignment. It can be seen from the evolutionary tree that *TcCAD2*, *TcCAD5*, *TcCAD11*, *TcCAD19*, *AtCAD4,* and *AtCAD5* are in the first class and belong to the same group (I). *AtCAD4* and *AtCAD5* have been proven to be related to the synthesis of lignin; therefore, *TcCAD2*, *TcCAD5*, *TcCAD11,* and *TcCAD19* were chosen as target genes for further studies to investigate their functions in the phenylpropanoid biosynthesis pathway (Figure 12).

## 3. Discussion

Valuable broad-leaved species have become a scarce resource, particularly those with a high wood hardness; high density; attractive appearance, color, and texture; and special craft properties suitable for producing high-end furniture, musical instruments, handicrafts, as well as other high-end products [19]. At present, the forest resources in China are limited, and the existing forest stands are of low quality and are unevenly distributed, but the demand increases year by year. Forests are used not only for wood and other industries but also to improve ecological and environmental resources [20]. The selection and promotion of valuable broad-leaf species can satisfy the demand for particularly valuable wood and improve the value of forests. With the short supply of high-end wood, *T. ciliata* (which has excellent material properties and an attractive texture) represents an alternative precious wood species that can be used efficiently and has high economic, ecological, and social value. The color of wood is an important indicator to assess wood quality and its commercial value. There are significant differences in the wood color of *T. ciliata* of different provenances. Studies have demonstrated that the differences in wood properties are mainly influenced by the type and content of the wood components. Wood is a natural polymeric compound; from a chemical point of view, wood is mainly composed of cellulose, hemicellulose, lignin, and extractives. Of these, lignin, extractives, and hemicellulose have the greatest influence on the wood color [10].

In this study, we identified the metabolites involved in lignin synthesis as well as the genes encoding these metabolites by conducting a joint transcriptome–metabolome analysis, providing deep insight into differences in wood quality, including wood color. The transcriptome results reveal that there are eight differentially expressed genes in the phenylpropane synthesis pathway (ko00940) in the non-red and red wood cores of *T. ciliata*, which are likely related to lignin synthesis. There are also three differentially expressed metabolites in the phenylpropane synthesis pathway (ko00940) in both the red and non-red wood cores, including p-coumaryl alcohol, coniferin, and sinapinaldehyde. These can directly influence lignin synthesis, which contributes to different wood colors. The combined analysis of the integrated transcriptome and metabolome presented three differential genes (Tci03G002560, Tci02G000070, and Tci09G002190) associated with two metabolites (coumestrol and pinocembrin). We therefore hypothesized that the wood properties of the *T. ciliata* samples may be determined by their lignin content. We subsequently measured the lignin in the wood of different wood-color samples. The results reveal that the wood with different wood properties varies in the lignin content. The lignin content of the wood cores with darker wood colors is significantly higher than that of those with lighter wood colors, proving that the wood properties of *T. ciliata* are related to the lignin content. This also confirms the results obtained from the transcriptome and metabolome analyses of the wood of *T. ciliata*.

Following the metabolome and transcriptome analysis, the lignin content of *T. ciliata* wood with different wood colors was measured. The results show that the lignin content of *T. ciliata* wood varies considerably with its wood color, with the lignin content of the dark-colored wood core significantly higher than that of the lighter-colored one. This suggested that the wood color of *T. ciliata* might be related to the content of lignin, which agrees well with the results obtained from the transcriptome and metabolome analyses of *T. ciliata* wood.

The biosynthesis of lignin is an extremely complex process that initiates with phenylalanine. It undergoes a series of enzymatic reactions to form coumarin (p-coumaryl alcohol), mustard alcohol (sinapyl alcohol), and coniferyl alcohol [21] prior to a complex polymerization reaction with the help of various enzymes [22,23,24] to form three lignin monomers (G-lignin, H-lignin, and S-lignin). Among these, 35 different monomers have been identified to date [23]. The lignin synthesis pathway mainly involves eight structural genes, among which PAL, C4H, and 4CL are the pre-structural genes for lignin biosynthesis; COMT and F5H are the genes for G-lignin and S-lignin biosynthesis. Cinnamyl alcohol dehydrogenase (CAD) is one of the rate-limiting enzymes in the whole synthesis pathway [25,26]. Since the discovery of the first *CAD* gene in tobacco [27], *CAD* studies have been conducted on pomegranate and cotton [28,29]. The *CAD* gene family can be divided into three subclasses based on their homology and affinity to substrates [30]. Subclass 1 plays an important role in lignin biosynthesis; subclasses 2 and 3 belong to multi-substrate alcohol dehydrogenases [31,32], which have a variety of physiological roles. The function of subclass 3 CAD proteins remains to be elucidated [33], and lignin biosynthetic pathways are also continually being revised and updated [34,35,36,37,38,39].

Tci09G002190 was identified as a *CAD* (cinnamyl alcohol dehydrogenase) gene through further analysis. It is a key enzyme in the lignin synthesis pathway and mainly acts in the final step of this pathway. Its activity and content can affect the synthesis of coumarin alcohol (p-coumaryl alcohol), from which the lignin monomers are synthesized. Therefore, Tci09G002190 (*TcCAD*) was chosen as the target gene for the subsequent bioinformatic and subcellular localization studies.

Differences in wood color and wood properties are affected by many factors. The composition of wood relies on a combination of factors and is not dominated by any single one. Growth environments can also affect wood properties. Further in-depth research is required to fully explore the origins of varieties in wood properties and comprehensively analyze their influencing factors.

## 4. Materials and Methods

### 4.1. Plant Material

**Figure 13:**
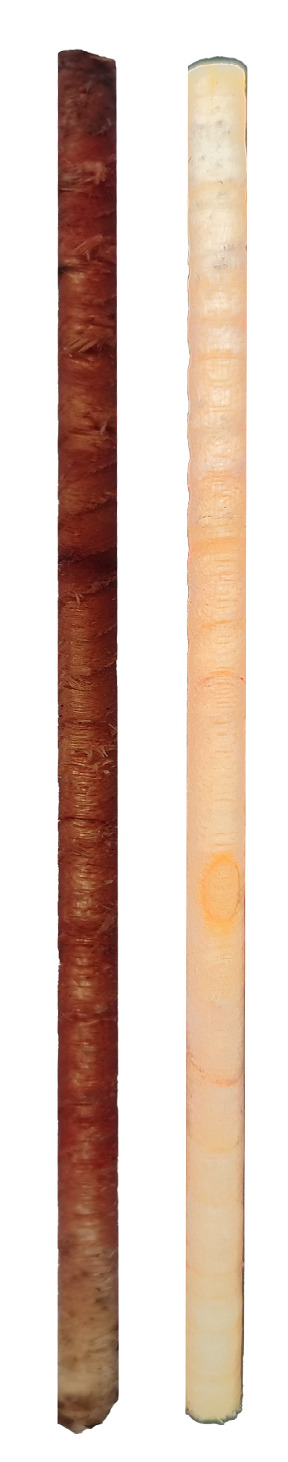
The *T. ciliata* wood cores were of different provenances. Note: On the left is a red wood core from Baoshan, and on the right is a non-red wood core from Australia.

We used ten-year-old *T. ciliata* specimens of different provenances, which were planted at the Zengcheng Experimental Base of the South China Agricultural University. One was from Baoshan, Yunnan Province, China (named No. 61), and the other was from Kangaroo Valley, New South Wales, Australia (named No. 11). The wood characteristics of the two provenances were quite different, especially the wood color (Figure 13). The wood core of each provenance was divided into heartwood and sapwood, resulting in four groups (X-61, B-61, X-11, and B-11). The metabolic group had six replicates per group for a total of 24 samples, and the transcription group had four replicates per group for a total of 16 samples. The weight of each sample was between 10 and 15 g. The core was quickly separated into heartwood and sapwood and cut into 1 cm (0.3–0.5 g) pieces. Small sections of the samples were collected in a multi-directional manner, mixed, and evenly distributed. The samples were wrapped in tin foil and quickly frozen in liquid nitrogen at −80 ℃ for the subsequent transcriptome sequencing and metabolite extraction. There were four replicates used in the transcriptomic analysis and six replicates used in the metabolomic analysis. The two analytical materials overlapped.

### 4.2. Metabolite Determination

The 24 samples were subjected to a metabolomic analysis at Beijing Bemac Biotechnology Co., Ltd. (Beijing, China). A quality control analysis was performed based on the metabolomic data obtained from the experimental design, sample collection and processing, metabolite extraction, and analysis. This allowed for the screening of differential metabolites as well as the prediction and analysis of their function in the samples. The liquid-mass spectrometry system used for metabolomics analyses consisted of an Acquity I-Class PLUS ultra-high-performance liquid chromatography tandem with an AB Sciex Qtrap 6500+ high sensitivity mass spectrometer, The column used was an Acquity UPLC HSS T3 column (1.8 μm 2.1 * 100 mm) (Waters, Milford, MA, USA). The screened differential metabolites were functionally annotated using the KEGG and GO databases to obtain the metabolic pathways involved.

### 4.3. Transcriptome Sequencing

High-purity RNA was extracted and detected according to the method reported by Song et al. [3]. The library construction and sequencing were performed according to the manufacturer’s instructions at the same institution where the metabolomic was analyzed. Eukaryotic mRNA was enriched using magnetic beads with oligo (dT) primers. The mRNA was then randomly interrupted by adding a fragmentation buffer. The first cDNA strand was synthesized using mRNA as a template with six-base random primers. The second cDNA strand was synthesized by adding a buffer, dNTPs, RNase H, and DNA polymerase I in a buffered solution. The cDNA was purified using AMPure XP beads. The purified double-stranded cDNA underwent end repair, the addition of an A-tail, and ligation of sequencing junctions. The fragment-size selection was ascertained using AMPure XP beads. The cDNA library was obtained through PCR enrichment. Following the completion of the library construction, the effective concentration of the library (i.e., >2 nM) was accurately quantified using qPCR to ensure its quality.

The libraries were pooled and sequenced using the Illumina platform based on the target downstream data volume once the library passed the quality check(Illumina NovaSeq 6000 platform, San Diego, CA, USA).

### 4.4. Transcriptome Data Analysis

The downstream data underwent filtration to obtain clean data, which were then compared with a specified reference genome to obtain the mapped data. The mapped data were used for the library quality assessment, which included an insertion fragment length test and a randomness test. The mapped data were also used for a structural analysis, which included a variable splicing analysis, new gene discovery, and gene structure optimization. Subsequently, the data were used for an expression level analysis, which included a differential expression analysis, functional annotation, and the functional enrichment of differentially expressed genes based on the expression of the genes in different samples or groups of different samples.

### 4.5. Comprehensive Analysis of the Metabolome and Transcriptome

A principal component analysis (PCA) was used to compare trends in the metabolites. Differentially expressed genes (DEGs) were identified using a *p*-value threshold of less than 0.05. Their functions and signaling pathways were enriched and analyzed using the KEGG database. Pearson’s correlation coefficients were calculated to determine the relationship between the genes and metabolites using R software (R version 4.2.3). Only DEGs and differentially expressed metabolites (DEMs) with coefficients of R^2^ > 0.8 were selected. The gene and metabolite datasets were analyzed using canonical correlation analysis (CCA) to reveal the interactions between the metabolites and genes in the cores of the *T. ciliata* samples and to construct a network.

### 4.6. qRT-PCR Validation

Ten differentially expressed genes, including upregulated and downregulated genes and the target gene (Tci09G002190) observed between the red and non-red wood cores, were screened and verified using real-time quantitative PCR. The TRIZOL method was used to extract the total RNA and synthesize the corresponding cDNA. The primers for the fluorescence real-time quantitative PCR were designed using Premier 5.0 software and were based on the representative sequences of the sequencing library. The main equipment used in the experiment was the Roche Light Cyler 480 system. The TUB gene was used as the internal reference gene. The expression levels of the target genes were calculated using the 2^−ΔΔCt^ method, with the expression level of the control group set to 1. This allowed for a comparison of the expression levels of each target gene with those of the control group.

### 4.7. Lignin Determination

*T. ciliata* wood core material for lignin extraction was consistent with that used for the metabolome and transcriptome. The samples were degreased by extraction with a ketone–alcohol mixture (acetone/ethanol = 2:1, *v*:*v*). Ten grams of the original samples were placed in a filter bag and extracted in a fume cupboard for 6 h until the extraction solution became colorless. The filter residue was placed in an oven at 60 °C to dry for 16 h, then cooled and weighed. An amount of 300 mg of the ketone–alcohol-extracted sample was accurately weighed into a 150 mL numbered reaction bottle. Then, 3 mL of 72% sulphuric acid was added while stirring constantly. The reaction bottle was placed in a water bath at 30 °C for 1 h, stirring every 5 min. After the water bath, 84 mL of deionized water was added to the reaction flask, and the flask was capped and placed in an autoclave (Yamato, Chongqing, China) at 121 °C for 1 h. After hydrolysis, the flask was removed and cooled to room temperature. The remaining residue in the reaction vessel was filtered through a crucible funnel to measure the acid-insoluble lignin content. All experimental determinations were conducted in triplicate.

(1) $$\mathrm{Klason}\;\mathrm{Lignin}\;(\%)\;=\;\frac{M_{1}-M_{2}}{M}$$

where M is the mass of the sample obtained by weighing (300 mg), M_1_ is the mass of the crucible funnel plus residue, and M_2_ is the mass of the crucible funnel. 

### 4.8. Phylogenetic Analysis of the TcCADs Gene Family in T. ciliata

The amino acid sequences of CAD proteins from T. ciliata and other species were used to construct a phylogenetic tree using the maximum likelihood (ML) method with MEGA7.0 software (http://www.bio-soft.net/tree/MEGA.htm, accessed on 6 December 2023), and 1000 replicates were set to calculate the bootstrap values to obtain the evolutionary status. The genes related to lignin biosynthesis were screened out as the main candidate genes for subsequent analyses of expression patterns and subcellular localization.

## 5. Conclusions

In this study, the lignin content of *T. ciliata* wood was measured using transcriptome and metabolome analyses for the first time. The metabolites related to lignin synthesis in the phenylpropanoid biosynthesis pathway (ko00940) and the genes related to the synthesis of this metabolite were mined using a combined transcriptome–metabolome analysis. Three genes related to lignin synthesis (Tci03G002560, Tci02G000070, and Tci09G002190) and two metabolites (p-coumaryl alcohol and coniferin) were identified. The correlation coefficients of the differential genes and differential metabolites were greater than 0.9 and reached a significant level, except that between Tci09G002190 and coniferin, and the correlation between Tci09G002190 and p-coumaryl alcohol was the most significant. We also identified the differential gene Tci09G002190 as a member of the *CAD* gene, which has previously been studied and observed to be associated with lignin synthesis. The lignin content is higher in the red wood cores of *T. ciliata* than in the non-red wood cores. In conclusion, greater attention can be given to the lignin synthesis mechanism in studies of wood properties of the precious tree species *T. ciliata*. This observation provides a solid basis for future research into the wood formation mechanisms of *T. ciliata*.

## Figures and Tables

**Table 1:** Differentially expressed gene results.

#ID	FDR	log2FC	Regulated
Tci25G012690	0.002352978	4.569650782	up
Tci04G002670	0.002322835	4.009840126	up
Tci28G006780	5.62 × 10^−7^	3.940942152	up
Tci24G013450	9.85 × 10^−5^	3.663677081	up
Tci18G013710	0.000243023	3.472814536	up
Tci17G009830	1.95 × 10^−5^	−5.371115339	down
Tci03G000040	4.31 × 10^−12^	−4.738548588	down
Tci24G007120	0.000123915	−4.351597937	down
Tci23G017350	0.006712827	−4.19246887	down
Tci03G006960	1.87 × 10^−7^	−4.10552472	down
Tci09G002190	5.47 × 10^−5^	−4.668484043	down

## Data Availability

The data presented in the study are deposited in the SRA repository in submission SUB14199550 (http://submit.ncbi.nlm.nih.gov/subs/sra/SUB14199550/overview, accessed on 6 December 2023); the accession numbers are SRR27848533, SRR27848532, SRR27848531, SRR27848530, SRR27848529, SRR27848528, SRR27848527, SRR27848526, SRR27848525, SRR27848524, SRR27848523, SRR27848522, SRR27848521, SRR27848520, and SRR27848519.

## Supplementary Materials

The following supporting information can be downloaded at: https://www.mdpi.com/article/10.3390/ijms25084541/s1.
