# Supplementary material for: The Integration of Transcriptome and Metabolome Analyses Provides Insights into the Determinants of the Wood Properties in Toona ciliata
Source: Int J Mol Sci. 2024 Apr 21;25(8):4541. doi: 10.3390/ijms25084541 (PMC11050501; doi:10.3390/ijms25084541)
Supplement: Supplementary file 1 [file ijms-25-04541-s001.zip › ijms-2907673-supplementary.pdf]

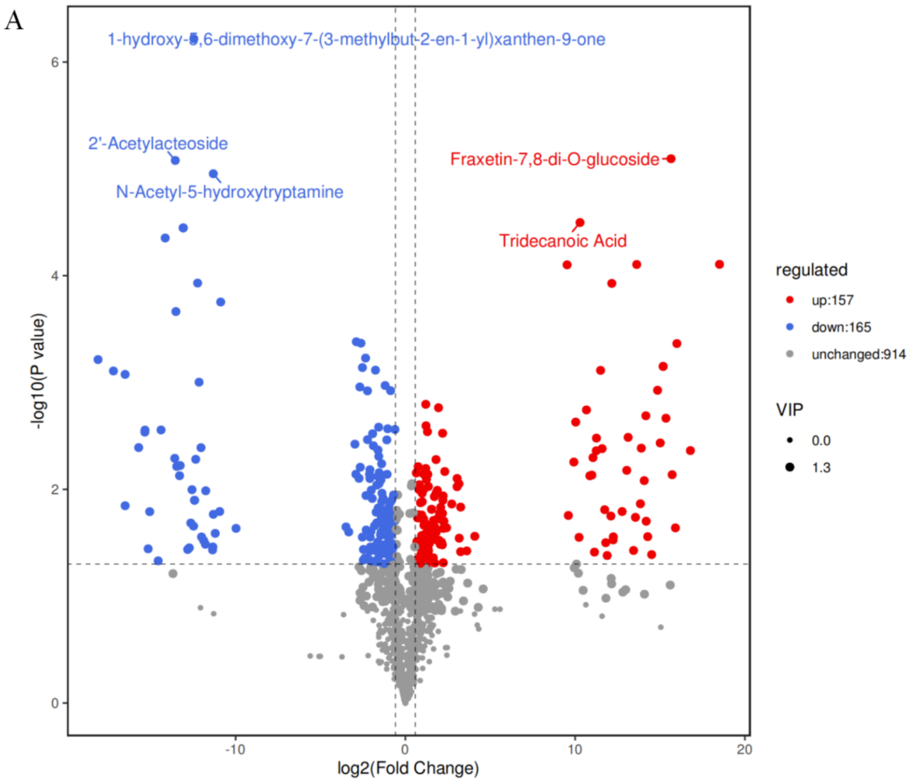

X-61 vs X-11

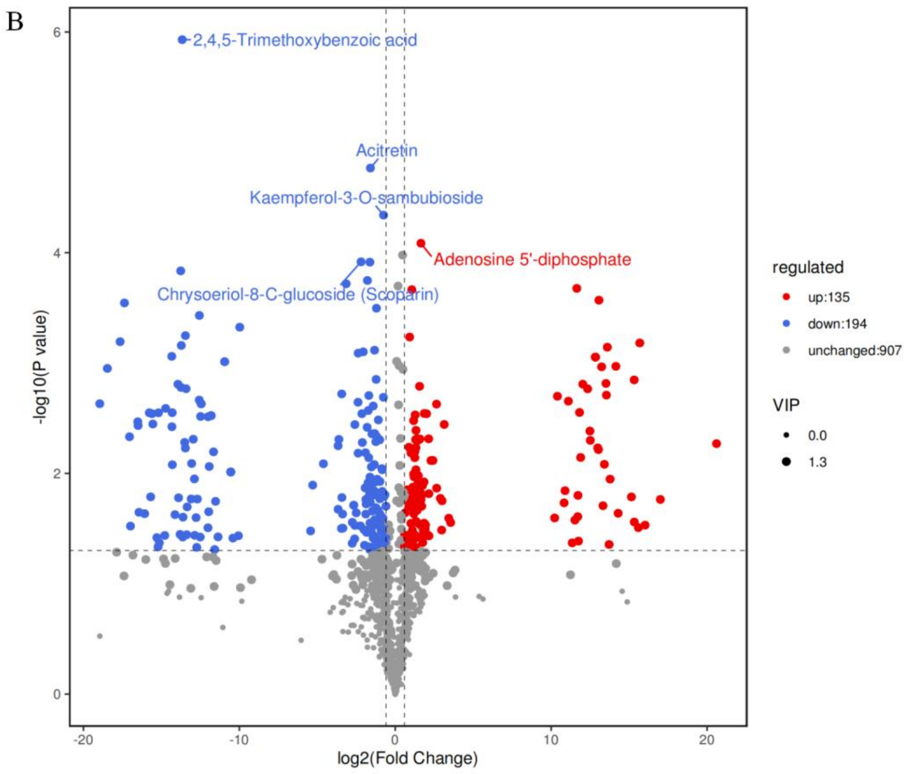

B-61 vs B-11

C

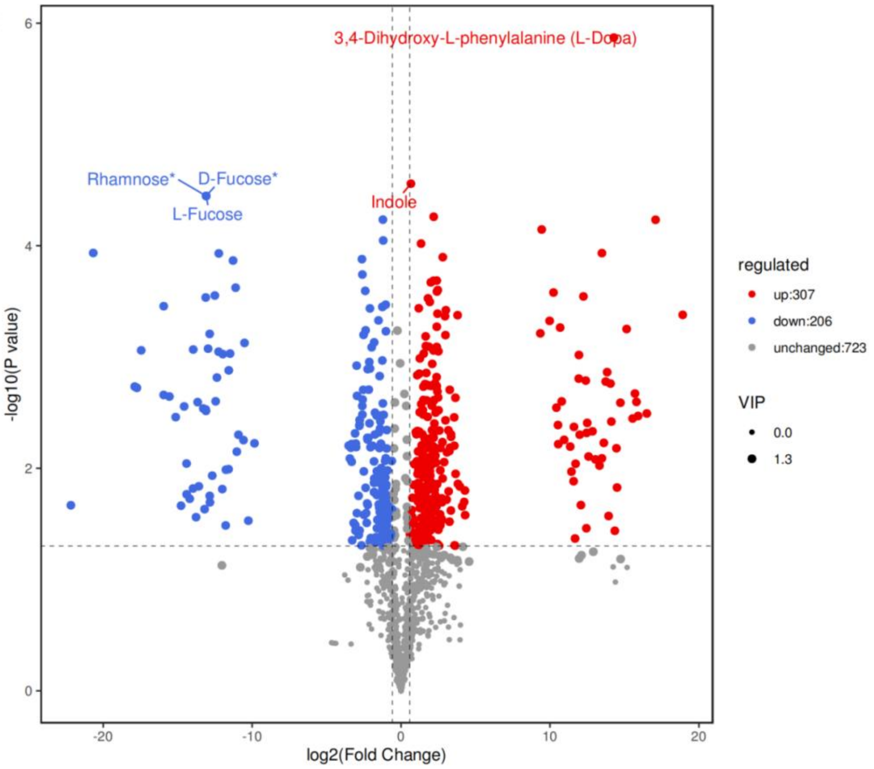

X-61 vs B-61

D

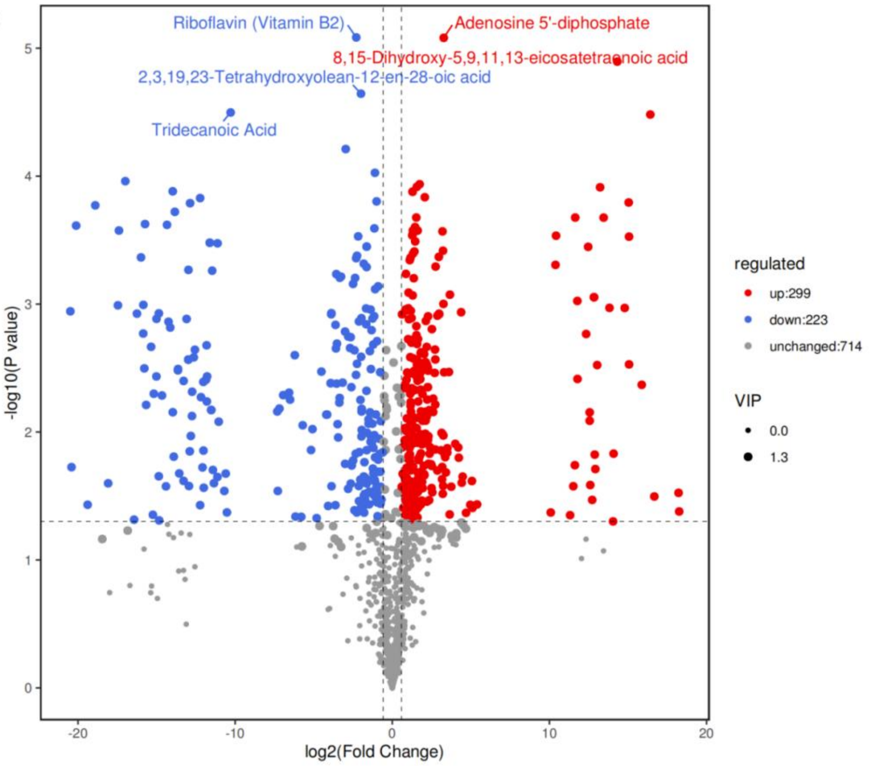

X-11 vs B-11

Figure S1. Differential expression volcano plots:
Note: A, B, C, D are the different groups, respectively. Each point in the volcano plot represents a metabolite, the x-axis represents the fold change of the group comparing each substance, the y-axis represents the P-value of the t-test, and the size of the scatter represents the VIP value of the OPLS-DA model, and the larger the scatter is the larger the VIP value is, and the more reliable the differentially expressed metabolites obtained by screening. The blue points in the figure represent down-regulated differentially expressed metabolites, the red points represent up-regulated differentially expressed metabolites, and the grey

points represent metabolites that were detected but the difference was not significant. In addition, the top 5 qualitatively detected metabolites were selected according to the P-value ranking and labelled in the figure.
